# Predictors of worsening TR severity after right ventricular lead placement: any added value by post-procedural fluoroscopy versus three –dimensional echocardiography?

**DOI:** 10.1186/s12947-021-00267-w

**Published:** 2021-11-21

**Authors:** Hoorak Poorzand, Mohammad Tayyebi, Sara Hosseini, Alireza Heidari Bakavoli, Faeze Keihanian, Lida Jarahi, Ali Hamadanchi

**Affiliations:** 1 Department of Cardiology, Echocardiologist, Vascular surgery Research center, Mashhad University of Medical Sciences, Mashhad, Iran; 2 Department of Cardiology, Electrophysiologist, Imam Reza Hospital, Faculty of Medicine, Mashhad University of Medical Sciences, Mashhad, Iran; 3 Department of Cardiology, Cardiologist, Faculty of Medicine, Mashhad University of Medical Sciences, first floor, Imam Reza Hospital, Shariati Square, Mashhad, Iran; 4 Department of Cardiology, Electrophysiologist, Ghaem Hospital, Faculty of Medicine, Mashhad University of Medical Sciences, Mashhad, Iran; 5 Department of Community Medicine, Faculty of Medicine, Mashhad University of medical sciences, Mashhad, Iran; 6 Department of Cardiology, Friedrich-Schiller University Hospital, Jena, Germany

**Keywords:** Pacemaker, Echocardiography, Three-dimensional echocardiography, Tricuspid regurgitation, Right ventricle

## Abstract

**Background:**

The effect of right ventricular (RV) leads on tricuspid valve has been already raised concerns, especially in terms of prognostic implication. For such assessment, three-dimensional transthoracic echocardiography (3D-TTE) has been used previously but there was no data on the use of post-procedural fluoroscopy in the literature.

**Methods:**

We prospectively enrolled 59 patients who underwent clinically indicated placement of pacemaker or implantable cardioverter defibrillator (ICD). Vena contracta (VC) and tricuspid regurgitation (TR) severity were measured using two-dimensional transthoracic echocardiography (2D-TTE) at baseline. Follow up 3D-TTE was performed 6 months after device implantation to assess TR severity and RV lead location.

**Results:**

Lead placement position in TV was defined in 51 cases.TR VC was increased after the lead placement, compared to the baseline study (VC: 3.86 ± 2.32 vs 3.18 ± 2.39; *p* = 0.005), with one grade worsening in TR in 25.4% of cases. The mean changes in VC levels were 1.14 ± 0.67 mm. Among all investigated parameters, VC changes were predicted based on lead placement position only in 3D-TTE (*p* < 0.001) while the other variables including fluoroscopy parameters were not informative.

**Conclusion:**

The RV Lead location examined by 3D-TTE seems to be a valuable parameter to predict the changes in the severity of the tricuspid regurgitation. Fluoroscopy findings did not improve the predictive performance, at least in short term follow up.

## Introduction

Due to prolonged life span of the community,the use of pacemakers and defibrillators is widely expanding, in order to control abnormal heart rhythms and prevent the symptoms of brady and tachyarrhythmia [1]. In addition, the indications of pacemakers’ implantation have been evolved over time. Moreover, in recent years, the emergence of ICD (implantable cardioverter defibrillator) and CRT (cardiac resynchronization therapy) in patients with arrhythmias and heart failure has increased the use of Cardiac Pacing [2]. One of the complications attributed to the presence of RV pacemaker lead is its interference with the tricuspid valve and tricuspid regurgitation (TR) [3]. It is still unclear whether the ventricular pacemaker lead can cause or exacerbate TR [4–6]. TR severity higher than moderate increases mortality and decreases survival in patients [1, 6]. Most of the studies conducted so far have considered the mechanical interference of the pacemaker lead with the tricuspid valve leaflets (cusp perforation, lead adhesion or Impingement) as the main cause of TR incidence in patients with pacemaker lead in RV [6]. Few studies have suggested that RV geometric change due to RV pacing or RV dys-synchrony is the cause of TR [6].

Reports indicate that 3D TTE is superior to 2D echocardiography in investigating the anatomical abnormalities of the tricuspid valve and origin of TR. Additionally, most studies used 3D echocardiography to determine lead-induced TR. On the other hand, this affect has not yet been assessed by post-procedural fluoroscopy. Hence, the purpose of the current study was to evaluate the effect of RV lead placement on tricuspid valve, utilizing fluoroscopy in combination with 3D-TTE.

## Methods

### Participants

Between January 2016 and January 2017, we prospectively enrolled 59 patients referred for PM or ICD implantation to our department, at a tertiary-care teaching hospital in the east of Iran.

Patients underwent baseline 2D-TTE.

Those with suboptimal echocardiographic or fluoroscopic images or unstable clinical status were excluded from the study.

Baseline information of the patients was recorded, including age, gender, history of underlying diseases and cardiovascular risk factors. Then, patients underwent 2D TTE by by an echocardiologist at the Department of Cardiology. The patients underwent lead implantation by an experienced electrophysiologist. At the end of the procedure, a fluoroscopy in the anterior-posterior projection was recorded. The second echocardiography was performed 6 months later, using 3D TTE to evaluate the lead location, and 2D TTE to measure TR severity, by the same echocardiologist.

### Echocardiographic image acquisition

**Fig. 1:**
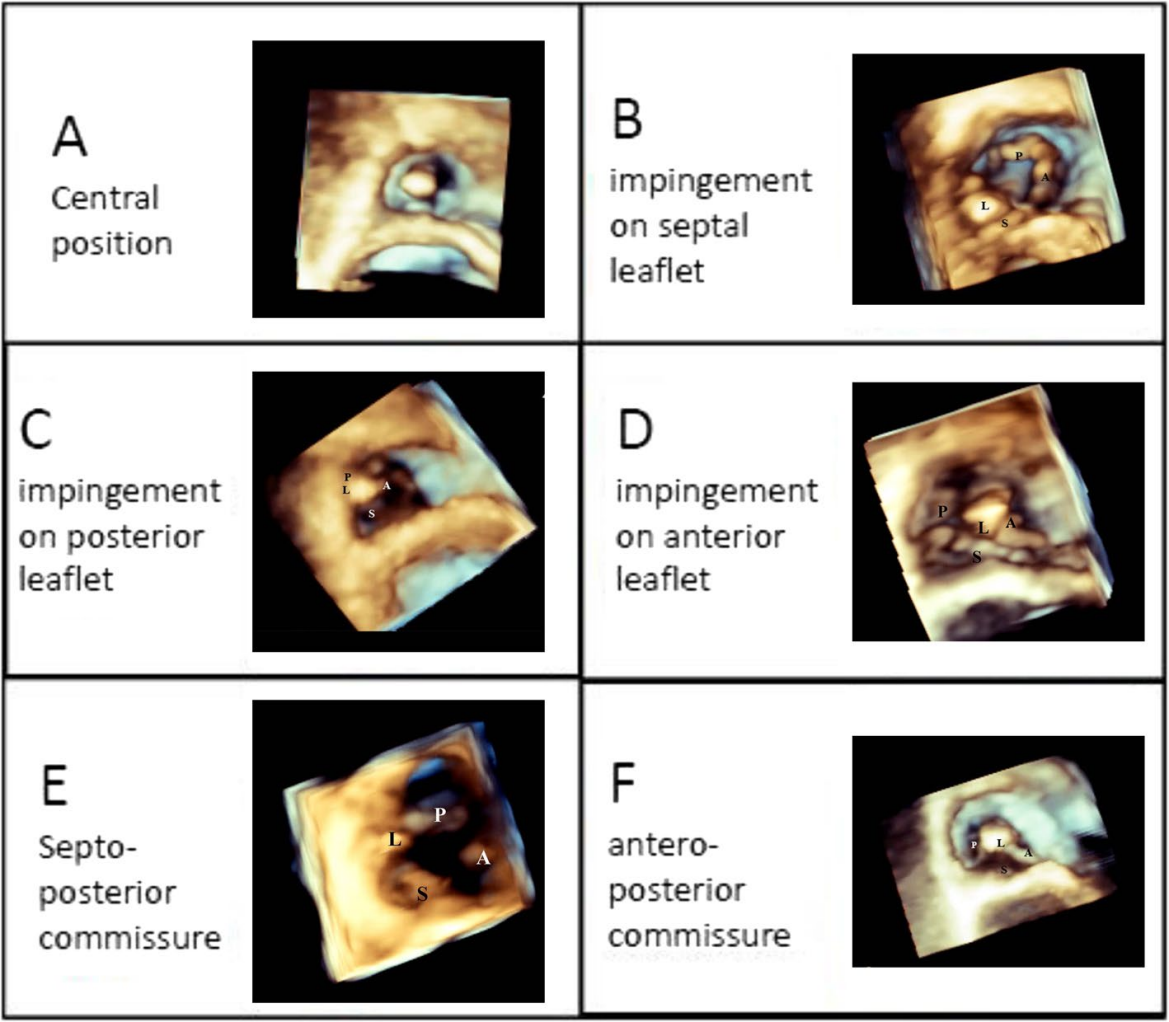
Lead location definition in 3D- TTE (**A** indicates anterior; L, lateral; P, posterior and S, septal). **A**) Central Position, **B**) Impingement on septal leaflet, **C**) Impingement on posterior leaflet, **D**) Impingement on anterior leaflet, **E**) Septo-posterior commissure, **F**) Antero-posterior commisure

Two and three dimensional echocardiography were performed using Philips IE33 Scanner (Bothell, WA, USA) with the Standard Probe matrix X5–1 by an Echocardiologist. During each echocardiographic study, the following parameters were measured and recorded: TR severity (using Vena Contracta diameter). RV function (by S Velocity and fractional area change), RV size (in mid RV diameter in RV focused view), RA size (as RA area in apical four chamber view), tricuspid annulus size, and the pacemaker lead tip position (in one of the three locations: apex, RVOT, septum). Tricuspid regurgitation was assessed again in the second phase of echocardiography (6 months after lead implantation). For 3D data acquisition, full volume or 3D zoom modes were used in the apical four-chamber view, with breath –hold and electrocardiographic gating over 4 consecutive cardiac cycles. After proper image optimization, tricuspid valve was evaluated in en face view to define the relation of the lead, while passing the valve. This relation was described as impingement on the septal, anterior, posterior leaflets, location at antero-posterior commissure, posterior-septal commissure, and anterior -septal commissure or centrally located (Fig. 1).

### Fluoroscopy

All patients underwent fluoroscopy post-procedurally in the catheterization laboratory with the X-ray system (Artis Zee, 150kv and 1000 MA severity, Siemens device). The imaging was performed in the anterior-posterior view (AP), and a short cine-angiography was recorded during one heart cycle. The digitally saved images were reviewed offline to determine different parameters: the tip position of the RV and LV leads, the curve length, the entrance point into the tricuspid ring, the angle and mobility of the RV lead.

**Fig. 2:**
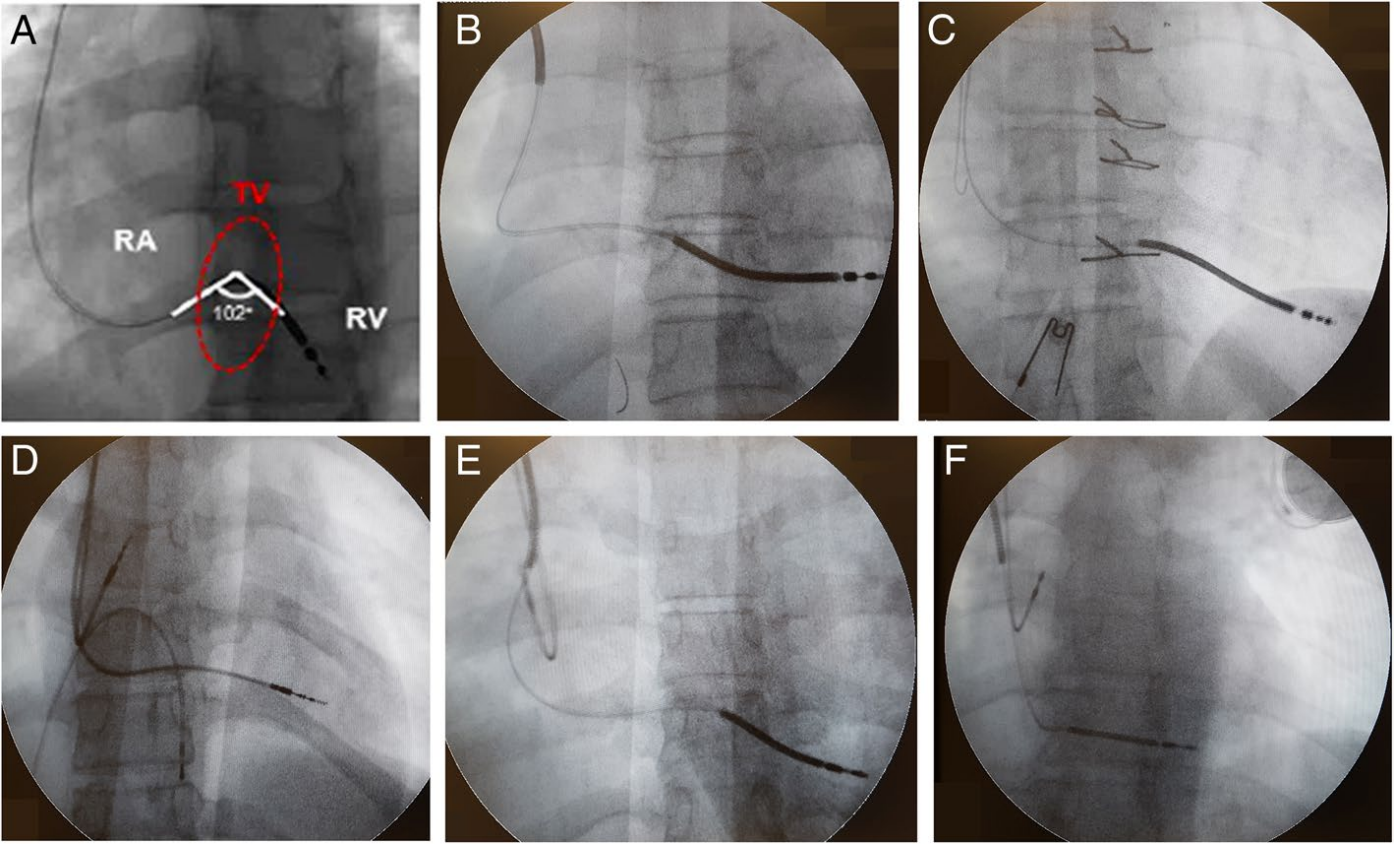
Fluoroscopic parameters. **A** RV lead angle; **B** Large RV lead curve; **C** Medium RV lead curve; **D** High tricuspid ring entrance point; **E** Middle tricuspid ring entrance; **F** Low tricuspid ring entrance

The RV lead tip position was defined as RVOT, mid-septal or apical positions. In CRT cases, the LV lead tip position was defined as inferolateral, posterolateral, lateral, or anterolateral. The RV lead curve length was described as two sizes: large and medium. In the large size, the curve was much longer than the necessary amount needed for the normal lead movement during the respiration or heart movements. Whereas in the medium size, the curve length was as much as needed for the normal lead mobility in inhale and exhale and the heart movements (Fig. 2). In subgroup categorization, also the RV lead tip position was divided in two groups: apical or non-apical [7].

The RV lead entrance point into the tricuspid ring was divided into three groups of low, middle and high, based on the lead position while passing the tricuspid valve ring in the AP view. The angle of RV lead at the tricuspid valve entry was measured as the greatest angle that RV lead makes through the heart cycle while passing the tricuspid valve (Fig. 2).

Finally, the extent of the RV lead mobility was defined as high, moderate and low degrees. When there was an optimal amount of the lead movement at the valve entry, according to the motion of the valve leaflets it was assigned as moderate mobility. Excessive and more than expected lead movement across the tricuspid valve was considered as high mobility. Low RV lead mobility was defined as minimal or no movement of the lead through the heart cycle.

### Statistical analysis

The continuous variables were expressed as mean ± SD and categorical variables as frequency and percentage. The normal distribution of data was analyzed using Kolmogorov-Smirnov test with modified Lilliefors, and then the right statistical test was selected. The paired t-test or non-parametric test was used for comparing the pre- and post-operative gradient compression and VC. The predictive parameters of VC changes were evaluated using multivariate regression analysis. SPSS version 18 (Released 2009; PASW Statistics for Windows, Chicago: SPSS Inc.) was used to analyze the data. *P* value less than 0.05 was considered as significant.

### Ethical considerations

A written informed consent was obtained from each patient after complete explanation of the research objectives and methodology. The patients’ information was kept confidential until the end of the study, and all data were encoded into statistical software. This research project was approved by the Ethics Committee of Mashhad University of Medical Sciences (IR.MUMS.SM.REC.1394.158). The patients were fully supported by researchers in the event of any research-related complication, and subjects were free to withdraw their consent at any stage of the study.

## Results

### Background information

Fifty-nine patients were enrolled in this study. Lead location was visible by 3D TTE in 86.4% of patients. In those in whom the lead location was not detectable, 5 out of 8 had pacemaker insertion, one ICD and two cases, CRT. Therefore, the final analysis was performed on the findings of 51 patients (12 females and 39 males) with the mean age of 60.9 ± 14.6 years. The most common implanted device was ICD (57.6%). Demographic data are presented in Table 1. Lead placement characteristics by 3D TTE are shown in Table 2.

**Table 1:** Background information in patients under study

Background information		Frequency (percent)
Referral reason	ICD implantation for Primary Prevention	21(35.6)
	ICD implantation for Secondary Prevention	13(22.0)
	Pacemaker implantation (AVB)	5 (13.6)
	Pacemaker implantation(SAN dysfunction)	3 (5.1)
	CRT implantation	12 (23.7)
Medical History	Diabetes	17 (28.8)
	Hypertension	7 (11.9)
	Non-obstructive CAD	9 (15.3)
	Significant CAD	50 (84.7)
Atrial Rhythm	Sinus rhythm	47 (92.1)
	Atrial Fibrillation	4 (7.9)
QRS	LBBB	13 (25.7)
	RBBB	4 (7.8)
	IVCD	3 (5.8)
	Normal QRS	31 (60.7)
AV Block	First Degree	7 (11.9)
	Second Degree	0
	Third Degree	5(8.5)

**Table 2:** Characteristic parameters related to RV lead placement in patients understudy

	Subgroup	Frequency (percent)	VC changes after lead placement
Lead placement type	ICD (single and dual chamber)	34 (66.6)	–
	PPM (single and dual chamber)	5 (9.8)	–
	CRT (CRT-D or CRT-P)	12 (23.5)	–
RV lead tip position	Apex	44 (86.2)	0.79 ± 1.11
	septum	5 (9.8)	0.45 ± 0.81
	Outflow tract	2 (3.9)	1.43 ± 2.26
RV lead curve	Large	33 (64.7)	0.99 ± 1.12
	Medium	18 (35.3)	0.38 ± 1.14
RV lead relation to the tricuspid valve ring	Middle	29 (56.8)	0.61 ± 1.15
	Low	21 (41.2)	1.02 ± 1.14
	High	1 (1.9)	−2.00 ± 0.00
RV lead mobility state	Mid	33 (64.7)	0.62 ± 1.19
	Low	12 (23.5)	0.91 ± 1.07
	High	6 (11.7)	1.28 ± 0.68
Lead location in 3DECH	Central	22(37.3)	0.38 ± 1.03
	Anterior leaflet	2 (3.4)	1.75 ± 0.35
	posterior-septal Commissure	9 (15.3)	0.04 ± 0.25
	Septal leaflet	8 (13.6)	1.40 ± 0.89
	Posterior leaflet	5 (8.5)	2.55 ± 1.23
	anterior-posterior Commissure	5 (8.5)	0.84 ± 0.83

### The vena Contracta (VC) changes

Overall, VC levels in the echo after lead placement were increased compared to the echo before lead placement (VC: 3.18 ± 2.39 vs. 3.86 ± 2.32 mm; *p* = 0.005). The mean changes in VC levels after to before lead insertion was reported 1.14 ± 0.67 mm (− 0.4–2.5) (Table 3).

**Table 3:** Variation of TR severity in regard to VC changes based on the location of lead placement in 3DECH before and after lead placement

Lead position	Variation of TR severity in regrade to VC changes						*P*-value
	Mild (n)		Moderate (n)		Severe(n)		
	Before	After	Before	After	Before	After	
Septal leaflet	5	3	3	3	0	2	0.025
Anterior leaflet	1	1	1	1	0	0	0.317
Posterior leaflet	4	0	1	4	0	1	0.034
Commissure posterior-septal	6	6	3	3	0	0	0.317
Commissure anterior-posterior	3	2	1	2	1	1	0.655
Central	16	14	3	6	3	2	0.721
Total	35	26	12	19	4	6	

### VC changes in accordance with 3D TTE lead placement position

**Fig. 3:**
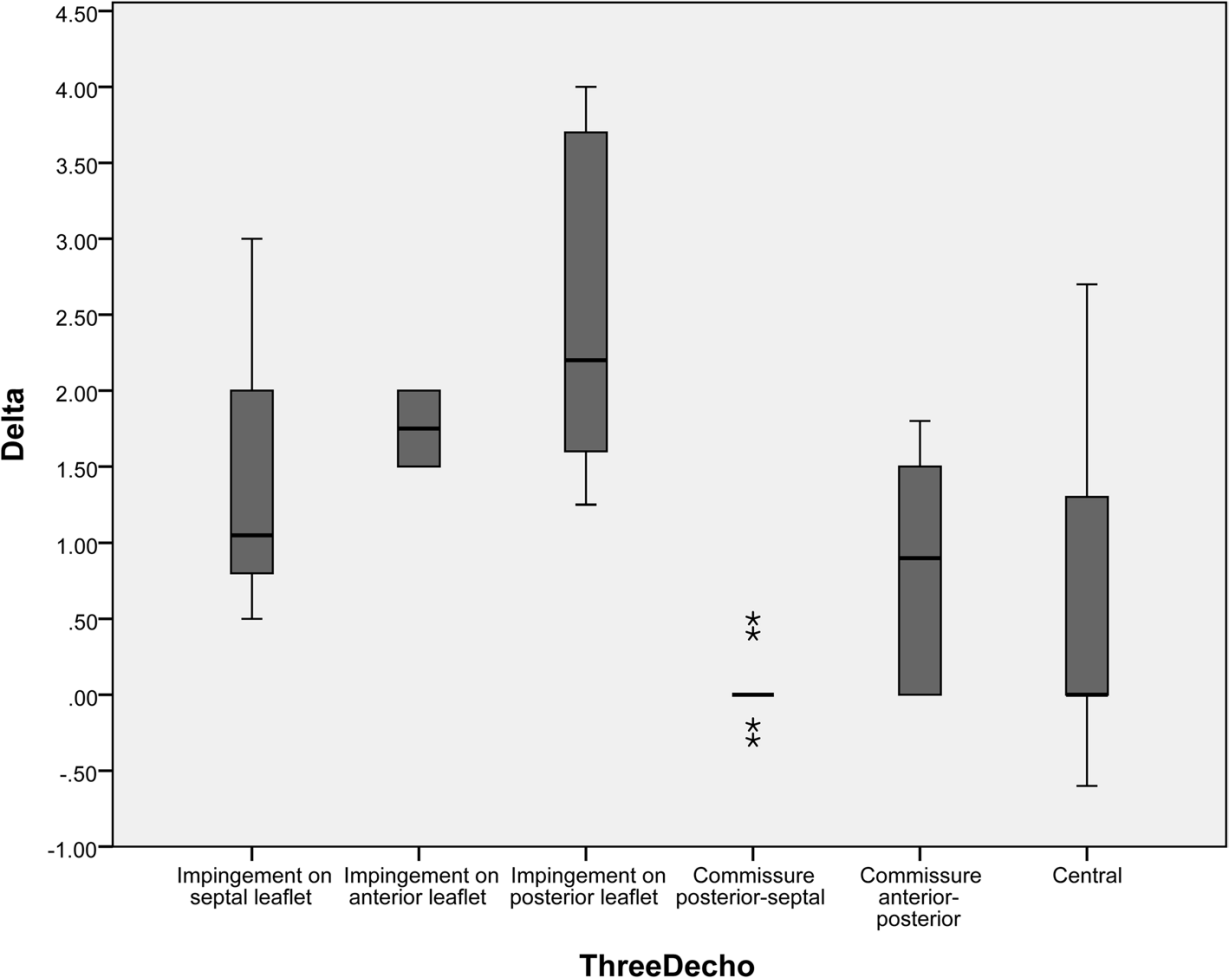
Comparison of the changes in TR severity (in regard to VC changes) according to lead location in 3DECHO, before and after lead placement

**Fig. 4:**
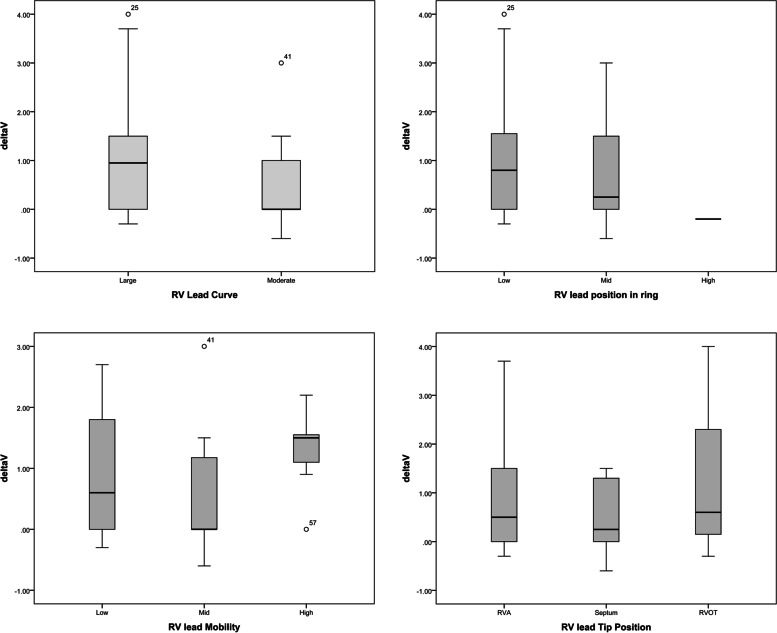
Comparison of the changes in TR severity according to RV lead tip position, curve, mobility and its spatial relation to TV ring

The mean changes in VC levels after lead insertion relative to before the insertion, showed the highest change in lead placement in the posterior leaflet and the least changes in central and posterior septal commissural position (*p* < 0.001). (Table 2, Fig. 3). Based on Wilcoxon statistical analysis, the VC changes were statistically significant in lead placement position in the septal leaflet (*p* = 0.025) and posterior leaflet (*p* = 0.034), but not statistically significant in the anterior leaflet (*p* = 0.317), posterior septal commissure (*p* = 0.317) and anteroposterior commissure (*p* = 0.655). Analysis of VC changes in different intervals showed that TR severity was mild (VC less than 3 mm) in 35 patients (68.6%) before lead placement, while it was mild in 26 patients (50.9%) after the placement. As well, TR severity was moderate (VC 3-7 mm) in 12 (23.5%) patients and severe (≥7) in 4 (7.8%) before lead placement, whereas it was moderate in 19 (37.2%) patients and severe in 6 (11.76%) after lead placement (Table 3). TR severity worsened one degree in 25.4% of patients, in which 10 cases (19.6%) changed from mild to moderate and in 3(5.8%) from moderate to severe. The latter group had ICD (two cases) or CRT (Fig. 4).

### VC changes in accordance with specifications of lead placement

The highest VC changes were observed in ventricular lead tip position in RVA (*p* = 0.744), low RV lead ring placement (*p* = 0.180), low RV lead mobility (0.136 = p), and large RV lead curve position (*p* = 0.139) (Table 2).

### The regression analysis

According to regression analysis, in the presence of variables, including age, gender, and 3D TTE lead placement position, VC changes were predicted based on the lead placement position in 3D TTE (*p* < 0.001), and the remaining factors were not significant. Additionally, in the presence of variables, including the RV lead mobility (*p* = 0.426), RV lead type (*p* = 0.309), lead in ring position (*p* = 0.399),lead tip position (*p* = 0.702), lead curve state (*p* = 0.071), and the 3DCH lead placement position, VC changes were predicted based on lead placement position in 3D TTE (*p* = 0.001) and the other factors were not significant. RV lead angle in fluoroscopy could not predict the increase in TR severity (*P* > 0.05). According to subgroup analysis, when we divided lead tip position into two categories of apical or non-apical, it was not predictor of TR severity (*P* > 0.05).

## Discussion

The aim of this study was to investigate the effect of RV lead placement on the tricuspid valve, using the data of 3D TTE and evaluating the probable added value of fluoroscopy. The most important results were: 1) the greatest change in VC was in lead impingement position on posterior and septal leaflets and the least change in postero-septal commissure and central position; 2) there was no difference between changes in VC before and after lead insertion, regarding lead tip position, RV curve position, RV lead ring position, and the RV lead mobility status; 3) considering the factors affecting TR severity (eg, RV lead placement types, and the variables evaluated during fluoroscopic and 3D TTE lead placement position), only 3D TTE lead placement position was predictive; and 4) risk factors for cardiovascular disorders and age had no association with VC changes following lead placement.

The findings of this study confirmed that TR severity increased following RV lead placement, using quantitative analyzes based on VC changes. The incidence of tricuspid regurgitation was about 30 and 50% before and after lead placement, respectively, with the new TR following lead placement found to be around 20%. In other studies, the incidence rate of tricuspid regurgitation after lead placement has been reported to be between 7 and 39% [6]. The differences in reported incidence rates, could arise from using different tools in determining the presence and severity of TR, different operators of echocardiography, and even using the data of physical examination as a criteria for assessing tricuspid regurgitation in some studies [8]. It should also be noted that most of the newly available information has been obtained from autopsy reports and surgical set; therefore, the study design and statistical sampling were clearly important factors affecting the results [6].

It is important to note that tricuspid regurgitation, as a mild physiological disorder, is considered a common finding in Doppler echocardiography, which can be seen in more than 80% of healthy people [9]. So, another big challenge would be TR exacerbation not only TR development. TR severity- based on VC –worsened from moderate to high in 5.8% and mild to moderate in 19.6%, after lead placement. In the study by Arabi et al. [10], the changes from moderate to severe was higher (19.5%). Lee et al. [11] also showed that TR severity increased following RV lead placement by two degrees (based on a six-point scale).

It was not important how to deal with lead placement relative to the tricuspid valve, but whether this could be related to the induction of high-severity tricuspid regurgitation after lead placement or not.

In our study, 15 cases (25.4% of cases) had lead interference with the leaflets, more prevalent for the septal and posterior ones, with the highest increase in TR VC,which was in line with the reports of Addetia et al. [12] .

### Predictors for worsening in TR severity

In the current study, the echocardiography and fluoroscopy variables including the 3D TTE lead placement position, RV lead tip position, RV lead curve position, RV lead ring position, RV lead mobility position alongside the known cardiac risk factors and age, were proposed as predictive parameters in a regression model. Among all the variables examined, only 3D TTE lead placement position could simultaneously predict the increase in TR severity following lead placement. Worsening in TR severity was related to the increase in mortality rate [13].

### Fluoroscopy versus 3d TTE

This was the first study that enrolled the data on fluoroscopy in an attempt to change the lead location, before ending the procedure of lead implantation, and to assess whether it could be used as a guide to reduce the lead-leaflet interference. But RV lead angle, curve or its mobility did not have a predictive role for worsening TR severity in this study.

3D TTE showed its promising role to define the undesired and high risk lead position, while passing TV, and could be beneficial for suggesting if the lead re-position might decrease such interference and prevent TR worsening. This strategy could be worthy to take, especially if diagnosed early, prior to the formation of fibrosis and inflammation leading to lead-leaflet attachment [13]. In those cases with the lead placement in high-risk situations of increased risk of TR worsening, shorter follow-up would also be considered in order to monitor the patient’s clinical condition.

## Limitations

This cohort included both patients with pacemaker and ICD implants, which was an important limitation of this study. We did not compare their results separately; though it could be hypothesized the ICD lead might have more interference with the leaflets and resultant more degrees of TR. This could be addressed in other studies with longer duration of follow-up.

In addition, no transesophageal echocardiography was used in our study, in which 3D echo data acquisition would be more accessible.

We just focus on the mechanical effect of the lead and did not consider other factors (eg, RV dyssynchrony after pacing), which could be a cause for exacerbating TR.

## Conclusion

Lead impingement on posterior and septal leaflets was associated with the highest change in vena contracta, while the lowest change was seen in posteroseptal and central position.

3D TTE could be employed to detect lead direction and location in the right ventricular and its effect on the induction of tricuspid regurgitation following RV lead placement and the related data could be used as predictive parameter. The lead angle, curve or its mobility in fluoroscopy did not show any additive value in this context.

### What’s new?

Fluoroscopy, in addition to 3D echocardiography, was used in this study to define whether it could be used to predict the effect of RV lead implantation on the induction of tricuspid regurgitation.

## Data Availability

The datasets used or analyzed in the current study are available from the corresponding author on reasonable request.
